# Development of rapid guidelines: 3. GIN-McMaster Guideline Development Checklist extension for rapid recommendations

**DOI:** 10.1186/s12961-018-0330-0

**Published:** 2018-07-13

**Authors:** Rebecca L. Morgan, Ivan Florez, Maicon Falavigna, Sergio Kowalski, Elie A. Akl, Kristina A. Thayer, Andrew Rooney, Holger J. Schünemann

**Affiliations:** 1 Department of Health Research Methods, Evidence and Impact and McGRADE Center, McMaster University Health Sciences Centre, Room 2C16, 1280 Main Street West, Hamilton, ON L8N 4K1 Canada; 2 Department of Pediatrics, University of Antioquia, Cra. 51D #62-29, Medellin, 050001 Colombia; 3 Hospital Moinhos de Vento, Porto Alegre, Brazil; 4 National Institute of Science and Technology for Health Technology Assessment, Universidade Federal do Rio Grande do Sul, Porto Alegre, Brazil; 5 Department of Internal Medicine, Division of Rheumatology, Universidade Federal do Paraná, R. Gen. Carneiro, 181, Curitiba, PR Brazil; 6 Department of Internal Medicine, American University of Beirut, Beirut, Lebanon; 7 National Center for Environmental Assessment (NCEA), Integrated Risk Information System (IRIS) Division, Environmental Protection Agency, Washington, DC United States of America; 8 Division of the National Toxicology Program, National Institute of Environmental Health Sciences, National Institutes of Health, Department of Health and Human Services, P.O. Box 12233, Mail Drop K2-02, Research Triangle Park, NC 27709 United States of America; 9 Department of Medicine, McMaster University, Health Sciences Centre, Room 2C14, 1280 Main Street West, Hamilton, ON L8S 4K1 Canada; 10 Cochrane Canada Center, McMaster University, Health Sciences Centre, Room 2C14, 1280 Main Street West, Hamilton, ON L8S 4K1 Canada

**Keywords:** Rapid guidelines, Guideline development, Clinical guidelines, GIN-McMaster Guideline Checklist

## Abstract

**Background:**

Practice guidelines require a substantial investment of resources and time, often taking between 1 and 3 years from conceptualisation to publication. However, urgent situations require the development of recommendations in a shorter timeframe. In this third and final article in the series exploring challenges and solutions in developing rapid guidelines (RGs), we propose guiding principles for the development of RGs.

**Methods:**

We utilised the Guideline International Network-McMaster Guideline Development Checklist (GDC) as a starting point for elements to consider during RG development. We built on those elements using the findings from a systematic review of guideline manuals, a survey of international organisations conducting RGs, and interviews of guideline developers within WHO. We reviewed initial findings and developed an intermediate list of elements, as well as narrative guidance. We then invited experts to validate the intermediate list, reviewing for placement, brevity and redundancy. We used this iterative process and group consensus to determine the final elements for RG development guidance.

**Results:**

Our work identified 21 principles within the topics of the Guideline International Network-McMaster GDC to guide the planning and development of RGs. Principles fell within 15 of the 18 checklist topics, highlighting strategies to streamline and expedite the guideline development process.

**Conclusions:**

We defined principles to guide the development of RGs, while maintaining a standardised, rigorous and transparent process. These principles will serve as guidance for guideline developers responding to urgent situations such as public health urgencies. Integration of these principles within currently disseminated guideline development standards will facilitate the use of those tools in situations necessitating RG recommendations.

## Background

Healthcare guidelines are statements that include recommendations intended to optimise healthcare, whether at the clinical, public health or health policy levels. They should be informed by a systematic review of evidence and an assessment of the desirable and undesirable consequences of alternative care options [1]. Practice guideline (PG) development can be a resource intensive and a time consuming process. The development timeframe of PGs vary across organisations, often ranging from 1 to 3 years [2–4]. These timeframes are not realistic for situations requiring immediate decisions and urgent recommendations, including situations of emerging infectious diseases, disasters and new evidence with a potentially vast health impact.

Organisations have adopted rapid guideline (RG) processes to shorten development timeframes. RGs can provide useful guidance and be conducted in an evidence-based and transparent manner [5]. One approach to expedite the guideline development process is to increase the resources (human or financial) and perform the standard amount of work in a shorter timeframe; unfortunately, however, increasing resources is not an option in most cases. A simple approach is to reduce the amount of work by narrowing the guideline scope to one or few recommendations, yet not many topics lend themselves to inform users sufficiently with single recommendations [6]. A further approach is to use shortcuts in the development process. The challenge is in identifying the shortcuts that have minimal impact on validity or credibility of the guideline produced rapidly. For example, using unsystematically identified evidence is likely to impact the validity and credibility of the guideline.

Guideline developers using shortcuts would need to maintain some ‘essential elements’ of the process to ensure highly credible recommendations. To date, there is no systematically developed guidance of which elements of the process are essential when developing RGs. In 2014, we developed the Guideline International Network (GIN)-McMaster Guideline Development Checklist (GDC) (http://heigrade.mcmaster.ca/home) [7], which is organised into 146 elements across 18 topics addressing all stages of the guideline enterprise from planning, to implementation and evaluation [7].

The objective of this article was to develop an extension of the GDC for RG development. This is the third and final article in the series on exploring challenges in developing RGs. In the prior two articles of this series, we systematically surveyed the current practices on RG development [8] and the perception of guideline developers at WHO about RG development processes [9].

## Methods

We adopted the WHO definition of RGs, as guidelines completed within a 1- to 3-month timeframe to provide guidance in response to an emergency, urgent need or new evidence [4, 10]. Interim guidelines refer to guidelines provided when new interventions, exposures or diseases arise, or new evidence becomes available or data are likely to be incomplete. Full guidelines provide complete coverage (e.g. surveillance, diagnosis, public health, and clinical interventions) of a health topic or disease. We use the classification included in that definition of the RG process throughout this series on RG development [8, 9].

We followed a four-step process to generate a list of guiding elements for RG development, involving (1) systematic survey of manuals and published RGs from international guideline development organisations [8]; (2) interviews to examine the perceptions and experiences of guideline developers at WHO [9]; (3) qualitative analysis of the results of the systematic survey and interviews; and (4) validation and alignment of the guiding elements with the GDC topics. Based on these previous studies [8, 9], the final qualitative analysis, and the GIN-McMaster GDC, we provide practical guidance for how to overcome challenges in RG development.

### Qualitative analysis of the results of the systematic review and interviews

We utilised the GDC as a starting point for elements to specifically consider in RG development [7]. One reviewer (RM) extracted an initial list of elements based on the results of the systematic survey and interviews into an Excel database. A second reviewer (IF) reread the documents and extracted additional elements. Reviewers included elements that identified gaps in published RGs or methods to improve, streamline, or standardise the RG development process. During this initial review, we focused on creating a comprehensive and inclusive list of RG-related elements. Reviewers organised these elements into exhaustive lists under the most relevant topics provided by the GDC [8, 9]. The reviewers discussed placement of elements within GDC topics to reach consensus.

### Identification of guiding elements

A small team (IF, RM, HS) then appraised the initial list of elements for order, brevity and redundancy, and prepared an intermediate table (Table 1). Elements included in the intermediate table reflected elements identified in the systematic survey as distinct to rapid guidelines, all distinct elements from qualitative interviews, and additional elements identified during the validation process.

**Table 1:** Intermediate table of elements identified from the systematic survey, interviews and validation process

GIN-McMaster GDC Topic	Elements identified from the systematic survey [7]	Elements identified from qualitative study interviews [8]	Additional elements identified during the validation process
1. Organisation, budget, planning and training	• Shortened timeframe for this work	• Define the amount of time allotted for the RG • Consider resources (time) needed for conducting the RG or standard systematic review • Consider resources (financial) needed for conducting the systematic review • Define the process for when resources (time and financial) are limited	• Have standard operating procedures specific for RGs in place and ready for use • Prepare training material appropriate for rapid training (e.g. online modules readily available for rapid viewing) • Have templates for RG ready for use • Identify peer reviewers early • Plan early for panel meeting • Is there a requirement or value to identify key data needs to support decision? The data needed to reach a decision may frame the project and question. For a chemical or other spill, the hazard or health effects are of great concern, but the first question may be to measure the extent of exposure (which will define some recommendations of the RG)
2. Priority-setting	• No distinct elements identified for rapid guidelines	• Address whether or not there is a need for an interim/rapid guidance • Define the rationale motivating the rapid as opposed to the standard development (e.g. new evidence about efficacy/cost-effectiveness/safety, emergent/dangerous situations, etc.)	• Identify and assess published guidelines addressing the same topic (might help in prioritising issues not covered by those guidelines)
3. Guideline group membership	• No distinct elements identified for rapid guidelines	• Consider involving a health economist • Involve representative from the clearance entity to expedite review and approval of final document	• Consider involving content experts with prior experience with guideline development methodology • Consider involving technical experts (systematic reviewers and methodologists) with prior experience with rapid reviews and RG methodologies; involve them early on • Organisation may develop a database of experts by area of expertise
4. Establishing guideline group process	• No distinct elements identified for RGs	• Consider virtual meetings • Use a mix of face-to-face and virtual meetings	• None
5. Identifying target audience and topic selection	• No distinct elements identified for RGs	• None	• Alert the target audience to the upcoming RG to increase engagement in development, review and uptake of the RG
6. Consumer and stakeholder involvement	• No distinct elements identified for RGs	• No elements identified for RGs	• Chemical spill importance to stakeholders
7. Conflict of interest considerations	• No distinct elements identified for RGs	• Exclusion from the guideline development group of participants reporting conflicts of interest	• Consider alleviating conflict of interest management-related restrictions when recruiting participants in a short timeframe is challenging
8. Question generation	• Consider a limited scope	• Consider a narrow scope	• None
9. Considering importance of outcomes and interventions, values, preferences and utilities	• Consider reducing the number of outcomes to a few critical ones	• Elicit values and preferences from qualitative literature	• Rely primarily on input of experts and stakeholders
10. Deciding what evidence to include and searching for evidence	• No distinct elements identified for RGs	• Define a systematic review process for when evidence is limited • Address exclusion criteria (e.g. grey literature, non-English language, etc.) • Base RGs on evidence from systematic reviews	• Identify and assess published systematic reviews addressing the same topic (might help in prioritising reviews for questions not covered by those systematic reviews) • Consider conducting rapid scoping reviews in preparation for the rapid review • Consider conducting rapid reviews
11. Summarising evidence and considering additional information	• No distinct elements identified for RGs	• None	• Rely on evidence solicited from experts to collect ‘additional information’ and identify relevant primary studies
12. Judging quality, strength or certainty of a body of evidence	• No distinct elements identified for RGs	• None	• None
13. Developing recommendations and determining their strength	• No distinct elements identified for RGs	• None	• Consider online meeting and pre-voting
14. Wording of recommendations and of considerations about implementation, feasibility and equity	• No distinct elements identified for RGs	• Finalise wording of recommendations during the panel meeting(s)	• None
15. Reporting and peer review	• Determine whether an expedited review process can be used for RGs	• Describe the review process, if it differs from PGs • Plan for a shorter review time	• Use pre-drafted templates for the final report, as well as automated reports produced by software like the GDT
16. Dissemination and implementation	• No specific elements identified for RGs	• Address potential obstacles for implementation	• None
17. Evaluation and use	• No specific elements identified for RGs	No specific elements identified for RGs	• None
18. Updating	• Define a date for when the RG will be conducted as a standard PG	• If providing interim guidance, define when the RG or full PG will be finished or conducted	• Emergent or dangerous situations may have a ‘staged release’ of RGs in the following order: (1) first action to protect public health, respond to crisis, spill that is heavily weighted to protect against worst-case scenario; (2) the second action or recommendation based on planned update based on new or additional information may recommend a change in values leading to less conservative recommendations

*PG* practice guideline, *GDC* Guideline Development Committee, *RG* rapid guideline

We changed the placement of some elements to different topics within the GDC. For example, we initially placed ‘Consider the resources (both time and financial) needed and available for conducting the systematic review’ within Topic 1: Organisation, Budget, Planning and Training; however, since this item refers specifically to the development of the systematic review used to inform the RG, we reordered it to Topic 10: Deciding what evidence to include and searching for evidence. Modifications to improve brevity and reduce redundancy among the list included grouping ‘Consider involving a health economist’ and ‘Involve representative from the clearance process to expedite review of final document’ within guidance for the composition of a guideline oversight committee. Similarly, if two or more elements represented the same theme, we combined them into one. For example, the interviews identified seven distinct rationales that might provide the impetus for developing RGs, namely (1) new evidence about efficacy; (2) new evidence about cost-effectiveness; (3) new evidence about safety; (4) pressure from country members of WHO; (5) the need to provide advice; (6) the need to respond to public opinion; and (7) emergent or dangerous situations (e.g. epidemic of an infectious disease, the management or control of biological, chemical or radioactive hazards). We recognised the overarching theme as clearly defining the motivation for developing an RG, which includes the situations listed previously. When we did not identify elements for any of the topics, we noted it in the table. For each element, we narratively listed the most closely relatable overarching topic from the GDC, and provided clarification and guidance to facilitate understanding and implementation in practice.

### Validation of elements

Using an iterative approach, we shared these elements and narrative descriptions with other authors (EA, KT, AR) to validate the assessments made. They assessed placement of the elements within the GDC topics, improving brevity and reducing redundancy, and suggested additional elements for inclusion in GDC topics. Additionally, they reviewed narrative guidance about RG elements and provided suggestions to increase clarity and utility when implemented into practice. We then described the overarching principles of RGs according to the GDC topics.

## Results

Of the 32 elements identified from the qualitative analysis of the results of the systematic review and interviews, we selected 21 discrete guiding principles for planning or developing RGs. We linked these principles in the topics identified in the GIN-McMaster GDC [7]. Table 2 lists the final principles for RG development identified from the systematic survey, interviews and validation process organised by the topics from the GDC.

**Table 2:** Final principles for the extension of the Guideline Development Checklist (GDC) for rapid guidelines (RGs)

Guideline Development Checklist for standard guidelines		Process modification
Topic	Description	Principles for RGs
1. Organisation, Budget, Planning and Training	*Organisation, budget, planning and training* involves laying out a general but detailed plan describing what is feasible, how it will be achieved and what resources are required to produce and use the guideline. The plan should refer to a specific time period, and be expressed in formal, measurable terms.	1. Define the amount of time available for development of the RG and the elements from the GDC that should be followed. 2. Develop RG-related standard operating procedures; develop templates for RGs; identify peer reviewers early on; and plan panel meetings as early as possible.
2. Priority-setting	*Priority-setting* is the identification, balancing and ranking of priorities by stakeholders. It ensures that resources and attention are devoted to those general areas (e.g. chronic obstructive pulmonary disease, diabetes, cardiovascular disease, cancer, prevention) where healthcare recommendations will provide the greatest benefit to the population, a jurisdiction or a country. A priority-setting approach needs to contribute to future plans while responding to existing potentially difficult circumstances (citations provided in checklist extension).	3. Define the rationale motivating the RG (e.g. new evidence about efficacy/cost-effectiveness/safety, emergent/dangerous situations, etc.). 4. Address whether there is a need for temporary and/or emergency guidance.
3. Guideline Group Membership	*Guideline group membership* defines who is involved, in what capacity, and how the members are selected for the guideline development and at other steps of the guideline enterprise.	5. Involve relevant individuals in the guideline oversight committee. 6. Develop a database of topic-specific experts by area of expertise to consult when establishing the guideline oversight committee.
4. Establishing Guideline Group Processes	*Establishing guideline group processes* defines the steps to be followed, how those involved will interact, and how decisions will be made.	7. When the timelines are short, greater emphasis should be placed on using virtual meetings (alone or along with face-to-face meetings).
5. Identifying Target Audience and Topic Selection	*Identifying target audience* involves describing the potential users or consumers of the guideline. *Topic selection* defines the topics to be covered in the guideline (e.g. diagnosis of chronic obstructive pulmonary disease).	8. Alert the target audience to the RG before release.
6. Consumer and Stakeholder Involvement	*Consumer and stakeholder involvement* describes how relevant people or groups who are not necessarily members of the panel but affected by the guideline, e.g. as target audience or users, will be engaged.	None
7. Conflict of Interest Considerations	*Conflict of interest considerations* focus on defining and managing potential divergence between an individual’s interests and their professional obligations that could lead to questioning of whether the actions or decisions are motivated by gain such as financial, academic advancement, clinical revenue streams or community standing. Financial or intellectual or other relationships that may impact an individual or organisation’s ability to approach a scientific question with an open mind are included.	9. RG guideline development panels may need a rapid process for implementing conflict of interest policies.
8. PICO Question Generation	*PICO question generation* focuses on defining key questions the recommendations should address, including the detailed population, intervention (including diagnostic tests and strategies) and outcomes that will be relevant for decision-making (e.g. should test A be used, or should treatments B, C, D or E be used in chronic obstructive pulmonary disease).	10. RGs should address a limited number of questions.
9. Considering Importance of Outcomes and Interventions, Values, Preferences and Utilities	*Considering importance of outcomes and interventions, values, preferences and utilities* includes integrating in the process of developing the guidelines, how those affected by its recommendations assess the possible consequences. These include patient and carer knowledge, attitudes, expectations, moral and ethical values, and beliefs; patient goals for life and health; prior experience with the intervention and the condition; symptom experience (for example, breathlessness, pain, dyspnoea, weight loss); preferences for and importance of desirable and undesirable outcomes; perceived impact of the condition or interventions on quality of life, well-being or satisfaction and interactions between the work of implementing the intervention, the intervention itself, and other contexts the patient may be experiencing; preferences for alternative courses of action; and preferences relating to communication content and styles, information and involvement in decision-making and care. This can be related to what in the economic literature is considered ‘utilities’. An intervention itself can be considered a consequence of a recommendation (e.g. the burden of taking a medication or undergoing surgery) and a level of importance or value is associated with that.	11. Outcome prioritisation process for each PICO should be brief. 12. Information on patients’ values and preferences can be informed by multiple methods, such as qualitative literature or patient advocacy groups.
10. Deciding what Evidence to Include and Searching for Evidence	*Deciding what evidence to include and searching for evidence* focuses on laying out inclusion and exclusion criteria based on types of evidence (e.g. rigorous research, informally collected), study designs, characteristics of the population, interventions and comparators, and deciding how the evidence will be identified and obtained. It also includes but is not limited to evidence about values and preferences, local data and resources.	13. Consider the resources (both time and financial) needed and available for when defining the process for conducting the systematic review. Scoping or rapid reviews may inform eligibility criteria and prioritisation.
11. Summarising Evidence and Considering Additional Information	*Summarising evidence and considering additional information* focuses on presenting evidence in a synthetic format (e.g. tables or brief narratives) to facilitate the development and understanding of recommendations. It also involves identifying and considering additional information relevant to the question under consideration.	14. Relevant primary studies and evidence solicited from experts may be used to inform ‘additional information’ in the evidence to decision table.
12. Judging Quality, Strength or Certainty of a Body of Evidence	*Judging quality, strength or certainty of a body of evidence* includes assessing the confidence one can place in the obtained evidence by transparently evaluating the obtained research (individual studies and across studies) and other evidence applying structured approaches. This may include, but is not limited to, evidence about baseline risk or burden of disease, the values and preferences, resource use (cost), estimates of effects and diagnostic test accuracy.	None
13. Developing Recommendations and Determining their Strength	*Developing recommendations* focuses on integrating the factors that influence a recommendation using a structured analytic framework, and a transparent and systematic process. *Determining the strength of the recommendations* refers to judgments about how confident a guideline panel is that the implementation of a recommendation exerts more desirable than undesirable consequences.	15. Use pre-meeting voting and virtual meetings to expedite the decision-making process.
14. Wording of Recommendations and of Considerations of Implementation, Feasibility and Equity	*Wording of recommendations* refers to choosing syntax and formulations that facilitate understanding and implementation of the recommendations. Such wording is connected to *considerations of implementation, feasibility and equity*, which refer to the guideline panel’s considerations about how the recommendation will be used and what impact it may have on the factors described.	16. Finalise the wording of the final recommendations during the panel meeting(s).
15. Reporting and Peer Review	*Reporting* refers to how a guideline will be made public (e.g. print, online). *Peer review* refers to how the guidelines document will be reviewed and how it can be assessed (e.g. for errors), both internally and externally, prior to its publication by stakeholders who were not members of the guideline development group.	17. Define and transparently record the process used when evidence is determined to be limited. 18. Expedited options for internal and external review of the RG should be explored, and if deemed possible, the process should be outlined in the RG.
16. Dissemination and Implementation	*Dissemination and implementation* focuses on strategies to make relevant groups aware of the guidelines and to enhance their uptake (e.g. publications and tools such as mobile applications).	19. RG implementation strategy should reflect the scope of the PICO. 20. RGs should outline and address any potential obstacles to implementation.
17. Evaluation and Use	*Evaluation and use* refers to formal and informal strategies that allow judgments about evaluation of the guidelines as a process and product; evaluation of the use and/or uptake; and evaluation of impact and whether or not the guideline leads to improvement in patient or population health or other consequences.	None
18. Updating	*Updating* refers to how and when a guideline requires revision because of changes in the evidence or other factors that influence recommendations.	21. When developing an interim guideline, the date for when the RG or full practice guideline will be conducted should be defined. If developing an RG, the date for when the full practice guideline will be conducted should be defined.

### Guiding principles

#### Principle 1 (Topic 1: Organisation, budget, planning and training)

Define the amount of time available for development of the RG and the elements from the GDC that should be followed.

The quality of the RG is influenced by several considerations about the organisation, budget and planning. If enough financial and human resources are available or could be mobilised, then the guideline developer should consider conducting the work expected for a full systematic review or guideline within an expedited timeframe. If financial or human resource constraints exist, guideline developers should be pragmatic and consider using abbreviated methods to meet the timeframe in which the RG is needed. A detailed protocol of the guideline (item 11 on the GDC) may be omitted when the organisation has established guideline methods in place. In the extreme case of an emergent or dangerous situation requiring an immediate response, the time constraints may define the type of RG possible.

#### Principle 2 (Topic 1: Organisation, budget, planning and training)

Develop RG-related standard operating procedures, develop templates for RGs, identify peer reviewers early on and plan panel meetings as early as possible.

Plan ahead to facilitate the RG-development process. Some aspects of the process that can be developed internally to prepare for situations that necessitate RGs include the development of standard operating procedures and templates for use. Peer reviewers of the final document can be identified early in the RG development process, as can the dates of the panel meetings. Both of these strategies allow for the coordination of schedules and may lead to greater availability of peer reviewers and panel member participation.

#### Principle 3 (Topic 2: Priority-setting)

Define the rationale motivating the RG (e.g. new and recommendation-changing evidence about efficacy/cost-effectiveness/safety, emergent/dangerous situations, etc.)

The developers should clearly state the rationale for why an RG is needed instead of a routine guideline. Suggested categories, based on findings from the systematic review and survey and interviews with WHO personnel [8, 9] for the rationale include (1) emergent and dangerous situations (e.g. epidemic of an infectious disease, the management or control of biological, chemical or radioactive hazards); (2) new and recommendation-changing evidence about safety; (3) new and recommendation-changing evidence about efficacy that could change current knowledge or practice; and (4) new and recommendation-changing evidence about cost-effectiveness. The latter reasons may require less need for RGs. However, pressure from communities or jurisdictions for rapid guidance may stem from any of the rationales listed above, or lead to the need for advice or response to public opinion. The involvement of all relevant stakeholders (Topic 3 on the GDC) will often not be feasible and requires abbreviated processes. In the case of an early response to an emergent issue (e.g. a spill), RGs are likely to be more conservative or health protective because of the overwhelming value of protecting public health in the short term (e.g. can justify removing people from their homes for a few days or weeks, but difficult to keep them out for months or years from a spill).

#### Principle 4 (Topic 2: Priority-setting)

Address whether there is a need for temporary and/or emergency guidance.

Decide whether interim guidance is needed before a RG becomes available. If yes, include the development of temporary guidance or rapid guidance in the planning documents. In emergency situations, an iterative process of following emergency or urgent guidance with a RG or full guideline may be required.

#### Principle 5 (Topic 3: Guideline group membership)

Involve relevant individuals in the guideline oversight committee.

Including a member of the institution’s clearance process in the guideline oversight committee to make sure that institutional requirements are met. If expertise is restricted to those involved in dealing with the emergency (e.g. human infection with the avian influenza virus), involve those experts early on and throughout.

#### Principle 6 (Topic 3: Guideline group membership)

Develop a database of topic-specific experts by area of expertise to consult when establishing the guideline oversight committee.

A database of external experts organised by area of expertise may expedite the identification of panel members and peer reviewers for the RG development process. Care should be taken to vary participation of identified external experts by RGs and continue to add to the database in preparation for future RGs.

#### Principle 7 (Topic 4: Establishing guideline group processes)

When the timelines are short, greater emphasis should be placed on using virtual meetings (alone or along with face-to-face meetings).

Virtual meetings may shorten the time needed for organisational planning. In addition, if cost is an issue, e.g. due to the urgency of the situation or existing organisational budgets, virtual meetings may provide an economical alternative. Virtual meetings may allow panels to meet more often and for a shorter time. However, virtual meetings may compromise the participation of some guideline panel members. Face-to-face meetings require logistics that can be handled rapidly, e.g. by established organisations.

#### Principle 8 (Topic 5: Identifying target audience and topic selection)

Alert the target audience to the RG before release.

During the RG development process, alerting the target audience to the upcoming RG may increase stakeholder involvement in the development, review, dissemination and uptake of the RG.

#### Principle 9 (Topic 7: Conflicts of interest (COI) considerations)

RG guideline development panels may need a rapid process for implementing COI policies.

Organisations with time consuming approaches to COI declaration and management might need to restrict panel membership to those not reporting direct financial COIs. However, when making RGs for urgent situations or on new interventions, participation from individuals with the most topic-specific expertise who have a financial or academic conflict may be unavoidable. The organisation would need to declare any modifications to their COI policies dictated by the need to conduct the RG.

#### Principle 10 (Topic 8: PICO question generation)

RGs should address a limited number of questions.

The development of PICO questions should reflect the resource limitations outlined in the planning process, including improving the precision of the PICOs and/or limiting the number of PICOs. A guideline with a limited number of questions would require fewer resources, by expediting the evidence review. Similarly, the scope of the guideline can be reduced by narrowing the target audience. It is important to establish an adequate process for question prioritisation in order to make sure the most appropriate questions are covered by the guideline. Distinguishing a single rapid recommendation from a guideline containing several recommendations to provide adequate coverage of a topic may be important. The former is suitable for very few situations, e.g. a new intervention for a narrow problem [6], while the latter often provides more practical, yet focused coverage of a topic, e.g. an emerging problem that may be addressed by multiple interventions [11, 12]. Guideline developers should document the processes of topic and question selection for the guideline to ensure transparency. In emergent or dangerous situations, information is needed to determine the extent of the research question to acutely focus the RG.

#### Principle 11 (Topic 9: Considering importance of outcomes and interventions, values, preferences and utilities)

Outcome prioritisation process for each PICO should be brief.

In addition to limiting the scope and number of PICOs, outcomes assessed to inform decision-making should be limited to include only those deemed critical, especially in situations where outcomes are informed by distinct systematic reviews. If outcomes assessed are reduced, make sure that they still address both benefits and harms. An iterative process is effective at developing the list of critical outcomes. First, panel members decide what information is needed to respond to the emergency, considering both the exposure and health effects. Based on the available information, determine the estimated time to collect missing information to inform the critical outcomes. In some emergent or dangerous situations, such as a chemical spill, some outcomes may be prescriptive given the information needed to inform decisions concerning the public.

#### Principle 12 (Topic 9: Considering importance of outcomes and interventions, values, preferences and utilities)

Information on patients’ values and preferences can be informed by multiple methods, such as qualitative literature or patient advocacy groups.

Patients’ values and preferences are crucial for the development of recommendations. Their point of view may be assessed through different strategies such as including patient member representatives in the discussion or performing systematic reviews of utilities. In emergency situations of chemical spills or outbreaks, the impacted population may be represented on the panel by a community member or spokesperson. Assessing patients’ values and preferences indirectly through published literature or guideline members’ opinion may be a time- and resource-saving strategy. In situations of RG, qualitative literature and the panel members’ surrogate values and preferences may be used if they are directly related to the relative importance of the outcomes that are considered critical for decision-making.

Flexibility of methods for ascertaining patients’ values and preferences may allow resources to be allocated for other steps of guideline development. Ensure that panel members have suitable clinical and field experience in order to adequately provide patients’ point of the view.

### Principle 13 (Topic 10: Deciding what evidence to include and searching for evidence)

Consider the resources (both time and financial) needed and available when defining the process for conducting the systematic review. Scoping or rapid reviews may inform eligibility criteria and prioritisation.

Similar to the process of resource consideration when determining the comprehensiveness of the RG, available time and labour will influence the quality of the systematic review. If time and budget constraints exist, consider abbreviated methods for updating existing systematic reviews, such as utilising previously published systematic reviews, rapid reviews or tailored search criteria to define smaller searches [13]. Performing scoping or rapid reviews on the RG topic may inform realistic objectives and eligibility criteria for the RG. In addition, these reviews may inform the prioritisation of topics for an RG or topics that could be considered later in the process.

RGs should be based on systematic reviews; however, the focus may be placed on identifying relevant existing, highly credible, and up-to-date systematic reviews with targeted updates as necessary. Similarly, previously published guidelines can be assessed for quality and either updated, if new evidence is available, or ‘adoloped’ (adopted or adapted) to the target environment [14]. ‘Adolopment’ describes an effective model to avoid redundancies in the guideline development process. Developers consider the potential of certain strategies in the following order: (1) verbatim adoption of the current guideline for the target setting; (2) adaptation of the guidelines for the target setting; or (3) de novo development of guidelines for situations in which guidelines either do not exist or are too indirect for application in the target setting [14]. For emergent or dangerous situations, if an existing systematic review is not available, ensure that the methods used to identify and appraise the evidence are transparently described [5]. Additionally, when the RG is iterative, clearly describe how further review of information impacts initial RG outcome or guidance.

In situations without relevant existing systematic reviews, developers should conduct rapid systematic reviews. When deciding what evidence to include and the process of searching for evidence, steps to reduce the amount of results, such as focused database searches, may be appropriate. For example, limiting the language of the article search to English may reduce the volume of articles to review, and time and resources to conduct translations. Similarly, developers can limit the search to only peer-reviewed articles instead of also including grey or unpublished literature.

#### Principle 14 (Topic 11: Summarising evidence and considering additional information)

Relevant primary studies and evidence solicited from experts may be used to inform ‘additional information’ in the evidence-to-decision table.

#### Principle 15 (Topic 13: Developing recommendations and determining their strength)

Use pre-meeting voting and virtual meetings to expedite the decision-making process.

Pre-voting on domain judgments within the evidence-to-decision table by panel members and synthesis of responses by the methodologist or subject-matter chair can identify consensus among panel members without any further need of discussion or dissonance requiring additional discussion. The strength and direction of recommendations can be decided using virtual panel meetings (e.g. with GRADE’s software GRADEpro, https://gradepro.org/).

#### Principle 16 (Topic 14: Wording of recommendations and of considerations about implementation, feasibility and equity)

Finalise the wording of the final recommendations during the panel meeting(s).

Finalising the wording of the recommendations during the panel meeting when the evidence is assessed and recommendations developed may streamline drafting the final document. Standardised wording to represent the strength and direction of the recommendations exists to facilitate this process [15]. Preparing draft recommendations by the organiser will reduce the time needed but requires buy-in by guideline panel members into the approach used.

#### Principle 17 (Topic 15: Reporting and peer review)

Define and transparently record the process used when evidence is determined to be limited.

To maintain transparency in the final document and inform subsequent iterations of RGs and development of the PG, developers should present details of the systematic review and evidence-assessment process.

#### Principle 18 (Topic 15: Reporting and peer review)

Expedited options for internal and external review of the RG should be explored, and if deemed possible, the process should be outlined in the RG.

Peer review of RGs is critical to the process; however, steps may be taken to expedite the process. For instance, by making arrangements and setting up deadlines with reviewers early on in the process of RG development. Transparency of the methods used for peer review should be maintained by documenting the process in the final document.

#### Principle 19 (Topic 16: Dissemination and implementation)

RG implementation strategy should reflect the scope of the PICO.

Similar to the precise definition of the target audience, the implementation strategy should reflect considerations of feasibility, focusing efforts on the target audience identified by the guideline scope.

#### Principle 20 (Topic 16: Dissemination and implementation)

RGs should outline and address any potential obstacles to implementation.

When describing dissemination and implementation in RGs, potential obstacles should be identified and addressed. These obstacles may require different strategies based on the objectives and rationale for the development of the RG. Examples of potential obstacles include the lack of availability of interventions, such as drugs or temperature-controlled supply chains required to maintain the efficacy of vaccines or treatment, in some countries.

#### Principle 21 (Topic 18: Updating)

When developing an interim guideline, the date for when the RG or full PG will be conducted should be defined. If developing an RG, the date for when the full PG will be conducted should be defined.

As part of outlining a strategy for how and when an update or a guideline revision will be needed, for interim guidance or RGs, a clearly defined timeline and date for when the full PG will be conducted should be provided in the document. This recognises that interim guidance and RGs are conducted under an expedited or consolidated process and additional evidence and thorough review may increase the certainty of the recommendation. In an emergent or dangerous situation, RG updates may be disseminated as ‘staged releases’ in the following order: (1) the first action/release is to protect public health, and respond to the crisis or spill that is heavily weighted to protect against worst-case scenario; and (2) the second release, based on new and additional information will address planned updates and change in values.

## Discussion

In this article, we summarise principles for the expansion of the GIN-McMaster GDC to the development of RGs. The principles are based on the GIN-McMaster GDC and informed by a systematic review of developed RGs and qualitative research [8, 9]. Guidance for development of systematic reviews in an abbreviated time without compromising rigor can be found in previously published literature [13]. Additionally, we recently described solutions to retain transparency and rigor in assessing the certainty of evidence when providing emergency, rapid or urgent guidance [5].

Apart from the information and the recommendations provided by the National Institute for Clinical Excellence [3], WHO [4] and the United States Centers for Disease Control and Prevention [16] handbooks, to date, there is no specific guidance of how to develop a RG nor a minimum set of elements that should be considered when doing so. In the prior articles of this series, we summarise the recommendations by these organisations and gained understanding of the perceptions about the development process and barriers from WHO developers [8, 9]. Therefore, we are confident that these results reflect the reality of the process and these elements will be useful and applicable in future processes.

The addition of the GIN principles for disclosing and managing COI make for a more comprehensive package of guideline development procedures and considerations [17]. These elements provide guidance for when urgent situations necessitate the development of guidelines in a shortened timeframe. These elements serve as the minimum set of standards (i.e. irreducible minimum of work) required and are intended for use by guideline developers to plan and track the process of RG development as a complementary tool or a manual for guideline development of the original Guideline 2.0 checklist, now called the GIN-McMaster GDC [7].

While full guidelines remain the gold standard in guideline development, RGs are often necessary to provide important evidence-based guidance in times of urgency and emergency. In emergent or dangerous situations, RGs may have unique considerations such as staged roll-out plans or involvement of different stakeholders. By building on the guiding principles for both the development of guidelines and disclosure of interests and management of conflicts in guidelines, these elements for the development of RGs will help in maintaining a systematic, rigorous and transparent process. Adoption and implementation of these elements encourages consistency and standardisation among researchers and organisations tasked with the development of RGs.

One key factor in the process of RG development is the conduct or use of a systematic review. This step is the core of any guideline development, and frequently one of the most time-consuming steps. In our analysis of the perceptions of RG developers in WHO [9], it was identified as the ‘Achilles heel’, because its quality could be negatively affected by the need to reduce time. Efforts to make the systematic review process more efficient while maintaining its quality should be part of any RG enterprise. Although recommendations exist about how and when to develop rapid reviews [13, 18, 19], this is still an area of further research, and improvements in this field will directly impact the RG process.

When developing recommendations in response to emergencies, it is challenging to reduce the time for development while maintaining methodological rigor, transparency, participative process and implementability of recommendations. Our principles may be useful for individuals and organisations with varying levels of experience in RGs interested in developing or implementing a systematic processes to develop recommendations in short time periods to respond to specific urgent scenarios. Having an available roadmap can make the decision-making process easier at the global, national, regional or local levels. In addition to its usefulness for the developer, this guidance provides an opportunity help policy-makers assess the completeness of a RG document that they intend to use.

While this paper focuses on the development of RGs, organisations could also consider using published guidelines, which would involve assessing those guidelines for their currency, quality, and relevance to the question(s) of interest. Based on this assessment, the organisation might decide to adapt as is, adolop or redevelop the guidelines [14]. Additionally, we do not yet address the development of emergency guidance, where timelines are too short to allow for conduct of systematic reviews of the literature. Such process could still benefit from some the principles described above such as using a systematic and transparent approach and involving experts on an emergency basis [5].

Similar to the principles of the GIN-McMaster GDC, the publication and dissemination of this extension for RGs will allow to solicit feedback from users to inform its revisions, updates and adaptations [14]. These reviews and revisions contribute to the continued validation of the checklist by confirming or refuting checklist elements. This represents an efficient way to gain insights about the properties of the GDC, as we did not identify other reference against which to validate.

### Strengths and limitations

The development of these principles is based on the results from a comprehensive systematic survey of guidelines and methods by the most influential organisations that produce RGs globally [8]. In addition, we conducted in-depth interviews with key RG developers at the WHO that allowed us to have a better understanding of their perceptions about RGs in the context of public health emergencies and special scenarios commonly faced by this organisation [9]. These two studies provided empirical evidence validating the development of these RG elements. In addition, evaluation of the preliminary checklist confirmed the inclusion and placement of items for the final RG development checklist.

Among the possible limitations, we should mention that we do not propose differential weight to the elements. Hence, we cannot indicate which elements are more important than others, and developers and readers should make those choices based on the context and their specific scenario.

## Conclusions

Our guiding principles represent a comprehensive list of considerations during the development of RGs and an expansion to the GDC. Although these principles aim to address all stages in the RG guideline process, there are several areas for which further guidance is needed. Additionally, this paper can be used as a set of principles by which to evaluate RGs relative to the standard process. Future work will focus on validating these elements, obtaining additional feedback from RG developers and keeping this checklist up to date.

## Abbreviations

COI: Conflicts of interest
GDC: Guideline Development Checklist
GIN: Guideline International Network
PG: practice guideline
RG: rapid guideline
